# Admission serum potassium levels and prognosis of vasospastic angina

**DOI:** 10.1038/s41598-021-84712-w

**Published:** 2021-03-11

**Authors:** Won-Woo Seo, Sang-Ho Jo, Sung Eun Kim, Hyun-Jin Kim, Seung Hwan Han, Kwan Yong Lee, Sung Ho Her, Min-Ho Lee, Seong-Sik Cho, Hack-Lyoung Kim, Sang Hong Baek

**Affiliations:** 1 Department of Internal Medicine, Kangdong Sacred Heart Hospital, Hallym University College of Medicine, Seoul, Republic of Korea; 2 Division of Cardiology, Department of Internal Medicine, Hallym University Sacred Heart Hospital, 896, Pyeongchon-dong, Dongan-gu, Anyang-si, Gyeonggi-do 14068 Republic of Korea; 3 Division of Cardiology, Department of Internal Medicine, Hanyang University College of Medicine, Seoul, Republic of Korea; 4 Department of Cardiovascular Medicine, Gil Medical Center, Gachon University, Incheon, Republic of Korea; 5 Department of Cardiovascular Medicine, Incheon St. Mary’s Hospital, The Catholic University of Korea, Incheon, Republic of Korea; 6 Department of Cardiovascular Medicine, Daejeon St. Mary’s Hospital, The Catholic University of Korea, Daejeon, Republic of Korea; 7 Division of Cardiology, Department of Internal Medicine, Soonchunhyang University Seoul Hospital, Seoul, Republic of Korea; 8 Department of Occupational and Environmental Medicine, Dong-A University College of Medicine, Busan, Republic of Korea; 9 Division of Cardiology, Department of Internal Medicine, Boramae Medical Center, Seoul National University College of Medicine, Seoul, Republic of Korea; 10 Department of Cardiovascular Medicine, Seoul St. Mary’s Hospital, The Catholic University of Korea, Seoul, Republic of Korea

**Keywords:** Cardiology, Interventional cardiology

## Abstract

Hypokalemia is a common electrolyte disturbance and is related to poor prognosis in patients with cardiovascular disease. However, the role of hypokalemia in patients with vasospastic angina (VSA) has not yet been studied. The present study enrolled 1454 patients diagnosed with VSA according to ergonovine provocation test results and available admission serum potassium data. The primary outcome was a composite of cardiac death, acute coronary syndrome, and new-onset life-threatening arrhythmia. Based on a hypokalemia definition as serum potassium concentration ≤ 3.5 mEq/L, the hypokalaemia group included 70 patients (4.8%). The median potassium levels were 3.4 mEq/L [interquartile range (IQR) 3.3–3.5] in the hypokalemia group and 4.1 mEq/L (IQR 3.9–4.3) in the no-hypokalemia group. The median follow-up duration was 764 days. Primary outcomes occurred in seven patients (10.0%) in the hypokalemia group and 51 patients (3.7%) in the no-hypokalemia group. The Kaplan–Meier analysis showed a higher cumulative incidence of primary outcomes in the hypokalemia group compared to that in the no-hypokalemia group (log-rank *P* = 0.014). Multivariate Cox regression analysis also showed that hypokalemia was an independent predictor of primary outcomes. In conclusion, hypokalemia at admission was associated with adverse clinical outcomes in VSA.

## Introduction

Hypokalemia is a common electrolyte disturbance in hospitalised patients and is often related to poor prognosis in patients with acute medical illness^1^. Although severe hypokalemia can cause life-threatening arrhythmia or cardiac arrest, most hypokalemia events are mild to moderate and identified during routine admission laboratory testing. Nevertheless, even mild to moderate hypokalemia is related to poor prognosis in patients with acute medical illness, particularly in patients with cardiovascular or chronic kidney disease^1,2^. Hypokalemia is an established predictor of poor prognosis in patients with heart failure^3^. In addition, lower potassium levels at admission are related to a higher incidence of ventricular arrhythmias or cardiac arrest in patients with acute myocardial infarction^4,5^.

Vasospastic angina (VSA) is a common cause of myocardial ischemia in east Asian patients^6^. In general, the long-term prognosis of patients with VSA treated with a vasodilator is relatively good; however, approximately 6% of patients experience adverse cardiovascular events such as cardiac death, myocardial infarction, or ventricular arrhythmia^7^. Hypokalemia causes vasoconstriction by the inhibition of Na–K pumps in vascular smooth muscle cells^8^. In addition, the extracellular potassium level is related to vascular endothelial function, vascular smooth muscle cell proliferation, and arterial thrombosis^2^. However, the role of hypokalemia on clinical outcomes in patients with VSA has not yet been studied. Accordingly, we evaluated the impact of hypokalemia as a prognostic factor in patients with VSA.

## Results

**Figure 1:**
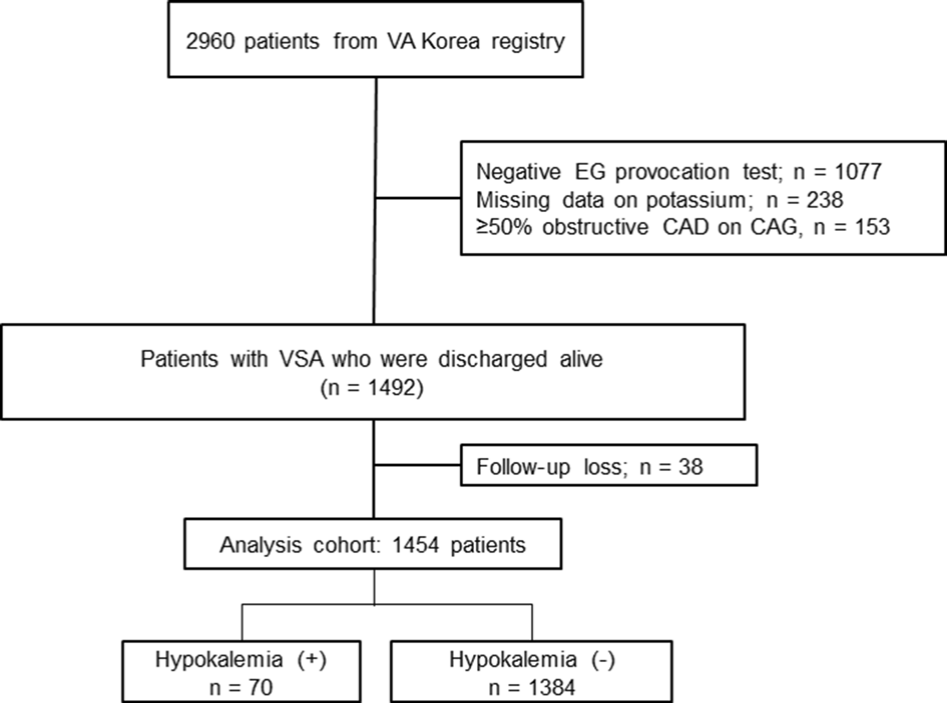
Study flow. *VA* variant angina, *EG* ergonovine, *VSA* vasospastic angina.

The VA-Korea registry included 2960 patients who underwent an ergonovine (EG) provocation test at 11 hospitals. Among them, 1883 patients were diagnosed with VSA based on positive test results on EG provocation tests. Of them, we excluded patients with missing data on serum potassium level at admission (n = 238) and ≥ 50% obstructive coronary artery disease on coronary angiography (n = 153). An additional 38 patients were excluded for loss to follow-up after discharge from index admission. Finally, 1454 patients were enrolled in this study, 70 (4.8%) of whom were included in the hypokalemia group according to serum potassium level at admission laboratory test (Fig. 1).

**Figure 2:**
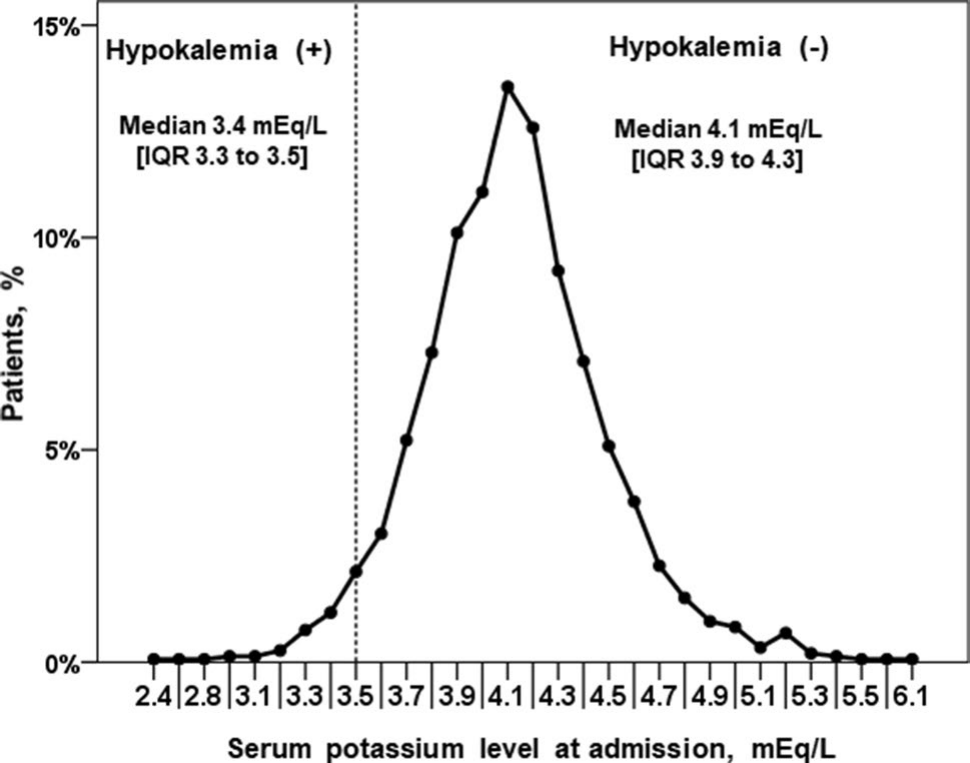
Distribution of serum potassium level at admission in the overall patient population.

The baseline characteristics of the study population are summarised in Table 1. Patients in the hypokalemia group had higher frequencies of hypertension, atrial fibrillation, prior thiazide diuretic use, and use of nitrate at discharge. Furthermore, hypokalemia group had lower hemoglobin, total cholesterol and potassium levels. The median potassium levels at admission were 4.1 mEq/L [interquartile range (IQR) 3.9–4.3] in the overall population, 3.4 mEq/L (IQR 3.3–3.5) in the hypokalemia group, and 4.1 mEq/L (IQR 3.9–4.3) in the no-hypokalemia group (Fig. 2). The multivariate logistic regression analysis showed that a history of hypertension and prior thiazide diuretic use were predictors of hypokalemia on admission (Table 2).

**Table 1:** Baseline characteristics of the patients with vasospastic angina.

	Hypokalemia (+) N = 70	Hypokalemia (−) N = 1384	*P* value
Age, years	55.4 ± 12.1	55.1 ± 1105	0.803
Men, n (%)	41 (58.6)	840 (60.7)	0.709
BMI, kg/m^2^	24.4 ± 3.1	24.8 ± 3.1	0.315
**Medical history**			
Hypertension, n (%)	36 (51.4)	507 (36.6)	0.016
Diabetes, n (%)	4 (5.7)	124 (9.0)	0.514
Dyslipidemia, n (%)	17 (24.3)	357 (25.8)	0.889
Chronic kidney disease, n (%)	9 (12.9)	135 (9.8)	0.409
Atrial fibrillation, n (%)	3 (4.3)	9 (0.7)	0.017
Peripheral artery disease, n (%)	0 (0)	1 (0.1)	1.000
Ischemic stroke or TIA, n (%)	1 (1.4)	20 (1.4)	0.892
**Medication at admission**			
Thiazide diuretics, n (%)	8 (11.4)	62 (4.5)	0.016
RAS inhibitor, n (%)	12 (17.1)	238 (17.2)	1.000
Calcium channel blocker, n (%)	21 (30.0)	264 (19.1%)	0.082
Beta-blocker, n (%)	4 (5.7)	100 (7.2)	0.803
Alpha-blocker, n (%)	0 (0)	8 (0.6)	0.736
Insulin, n (%)	0 (0)	5 (0.4)	1.000
Habitual drinking, n (%)	14 (20.0)	214 (15.5)	0.312
**Laboratory data on admission**			
Hemoglobin, g/dL	13.6 ± 1.5	14.0 ± 1.5	0.040
Creatinine, mg/dL	0.81 ± 0.26	0.81 ± 0.45	0.985
Total cholesterol, mg/dL	167.4 ± 38.9	176.8 ± 35.6	0.041
LVEF, %	63.4 ± 6.0	64.3 ± 5.9	0.239
Sodium, mEq/L	140.0 ± 2.6	140.0 ± 2.6	0.601
Sodium ≤ 135 mEq/L, n (%)	3 (4.3)	26 (1.9)	0.160
Potassium, mEq/L	3.36 ± 0.20	4.16 ± 0.33	< 0.001
**Ergonovine provocation test finding**			
Definite spasm, n (%)	41 (58.6)	761 (55.0)	0.623
Possible spasm, n (%)	29 (41.4)	623 (45.0)	0.623
Focal spasm, n (%)	22 (31.4)	553 (40.0)	0.169
Systolic BP on discharge, mmHg	124.3 ± 25.1	126.3 ± 17.8	0.516
Diastolic BP on discharge, mmHg	74.0 ± 16.1	77.1 ± 11.8	0.114
**Medication on discharge**			
Aspirin, n (%)	32 (45.7)	536 (38.7)	0.259
Statin, n (%)	32 (45.7)	662 (47.8)	0.807
Beta-blocker, n (%)	4 (5.7)	81 (5.9)	0.610
RAS inhibitor, n (%)	12 (17.1)	233 (16.8)	0.871
Calcium channel blocker, n (%)	62 (88.6)	1277 (92.3)	0.255
Nitrate, n (%)	65 (92.9)	1122 (81.1)	0.011
Alpha-blocker, n (%)	1 (1.4)	11 (0.8)	0.448

*BMI* body mass index, *TIA* transient ischemic attack, *RAS* renin-angiotensin system, *LVEF* left ventricle ejection fraction, *CKD* chronic kidney disease, *BP* blood pressure.

**Table 2:** Predictors of hypokalemia in patients with vasospastic angina.

Variable	Odds ratio	95% CI	*P* value
Age ≥ 60 years	0.70	0.39–1.24	0.222
Male	0.77	0.45–1.34	0.353
Hypertension	2.26	1.30–3.93	0.004
Diabetes	0.51	0.18–1.46	0.212
Chronic kidney disease	1.56	0.70–3.47	0.274
Habitual drinking	1.34	0.70–2.58	0.380
RAS inhibitor at admission	0.52	0.25–1.11	0.091
Thiazide diuretics at admission	2.80	1.15–6.84	0.024
Beta-blocker at admission	0.58	0.20–1.73	0.330
Hyponatremia	1.57	0.44–5.61	0.490
Definite spasm	1.14	0.69–1.88	0.601
Focal spasm	0.68	0.41–1.15	0.155

*RAS* renin-angiotensin system.

**Figure 3:**
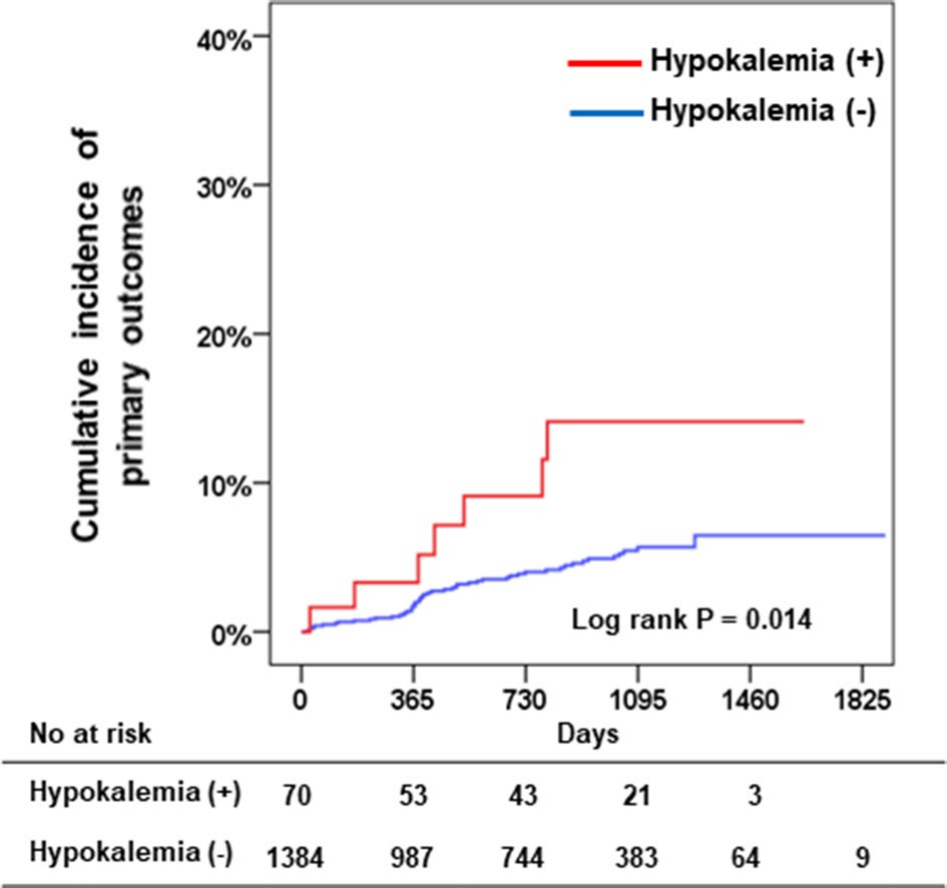
Cumulative incidence of primary outcomes between patients with and without hypokalaemia.

**Figure 4:**
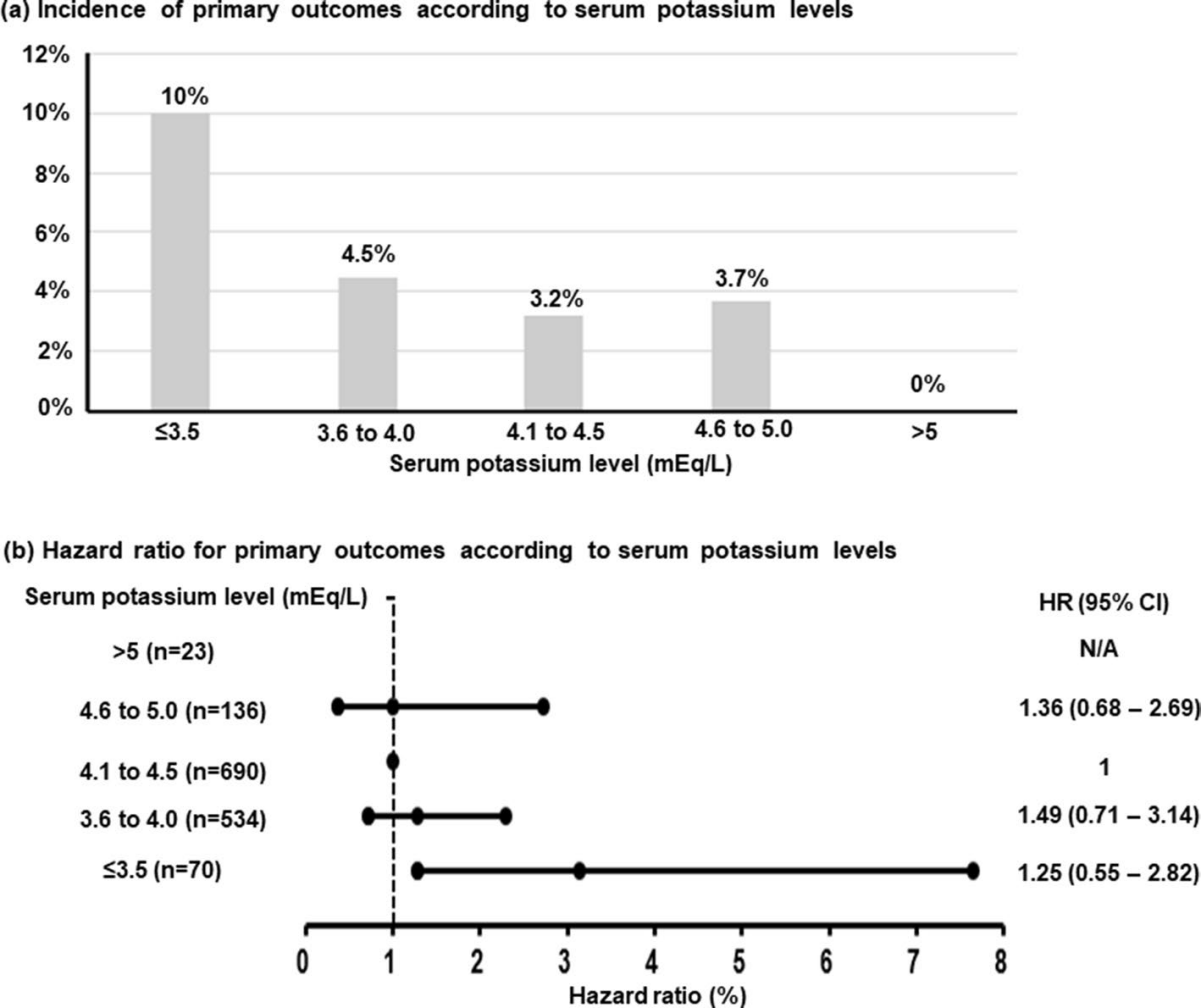
Relationship between admission potassium level and primary outcomes. (**A**) The incidence of primary outcomes was the highest in patients with potassium levels ≤ 3.5 mEq/L and the lowest in patients with potassium levels of 4.1–4.5 mEq/L. (**B**) Compared with the reference group (potassium level 4.1–4.5 mEq/L), the hazard ratio for primary outcomes was only significantly higher for patients with potassium levels ≤ 3.5 mEq/L.

The median follow-up duration was 764 days (IQR 347–1,103 days); primary outcomes occurred in seven patients (10.0%) in the hypokalemia group and 51 patients (3.7%) in the no-hypokalemia group (Table 3). The Kaplan–Meier survival analysis showed a higher cumulative incidence of primary outcomes in the hypokalemia group compared to that in the no-hypokalemia group (Fig. 3). Multivariate Cox regression analysis demonstrated that hypokalemia, along with diabetes, focal type of VSA, and hyponatremia, were independent predictors of the primary outcomes (Table 4). There was no event in patients with potassium levels > 5 mEq/L, except for this, incidence of primary outcomes was lowest in patients with potassium levels of 4.1–4.5 mEq/L (Fig. 4A). Compared to the reference group (potassium level 4.1–4.5 mEq/L) the hazard ratio for primary outcomes was significantly higher for the patients with potassium levels ≤ 3.5 mEq/L; however, no other significant differences were observed in the other groups (Fig. 4B).

**Table 3:** Incidence of primary outcomes in patients with vasospastic angina.

	Hypokalemia (+) N = 70	Hypokalemia (−) N = 1384	*P* value
**Total primary outcomes, n (%)**	7 (10.0)	51 (3.7)	0.019
Cardiac death, n (%)	2 (2.9)	8 (0.6)	
Acute myocardial infarction, n (%)	0	2 (0.1)	
Unstable angina, n (%)	4 (5.7)	34 (2.5)	
Ventricular tachycardia/fibrillation, n (%)	1 (1.4)	3 (0.2)	
Complete atrioventricular block, n (%)	0	4 (0.3)

**Table 4:** Multivariable Cox regression analysis for primary outcomes.

Variable	Hazard ratio	95% CI	*P* value
Age ≥ 60 years	1.23	0.67–2.23	0.500
Male	1.48	0.84–2.62	0.175
Hypertension	0.86	0.45–1.63	0.639
Diabetes	2.38	1.10–5.15	0.028
Chronic kidney disease	0.29	0.09–1.01	0.052
Habitual drinking	1.28	0.65–2.51	0.477
RAS inhibitor at admission	0.52	0.19–1.45	0.212
Thiazide diuretics at admission	1.31	0.40–4.24	0.653
Definite spasm (vs. possible)	1.20	0.69–2.07	0.523
Focal spasm (vs. diffuse)	2.22	1.31–3.75	0.003
Discharge aspirin	0.60	0.34–1.06	0.080
Discharge calcium channel blocker	0.90	0.35–2.35	0.836
Discharge nitrate	0.70	0.37–1.35	0.289
Discharge statin	1.04	0.61–1.78	0.893
Hyponatremia	6.45	2.32–17.96	< 0.001
Hypokalemia	2.89	1.27–6.56	0.011

*RAS* renin–angiotensin system.

## Discussion

The results of this multicentre cohort study showed that the prevalence of hypokalemia was 4.8% in patients with VSA and that hypertension and prior thiazide diuretic use were risk factors for hypokalemia. Furthermore, hypokalemia at admission was related to adverse cardiovascular outcomes in these patients.

Hypokalemia is a common electrolyte disturbance along with hyponatremia and is observed in approximately 20% of hospitalised patients^1,9^. Although severe hypokalemia can lead to life-threatening arrhythmia, rhabdomyolysis, respiratory failure, or death, most events in hospitalised patients are mild to moderate in degree. In this study, 4.8% of patients with VSA had hypokalemia and only one patient had severe degree (serum potassium < 2.5 mEq/L). The prevalence was relatively lower than those in other medical illnesses. Patients with VSA were less likely to have an electrolyte disturbance than those with other medical illnesses because they had fewer comorbidities, and VSA did not cause gastrointestinal or renal disturbances. In addition, while stress-induced β_2_-adrenergic stimulation related to intracellular potassium shifting has been reported in patients with severe medical illness^10,11^, patients with VSA are less affected by these mechanisms. Hypertension and prior thiazide diuretic use have been well-known risk factors for hypokalemia and were also consistently observed in the present study. Diuretics dose-dependently increase potassium excretion in distal tubule^12^. Cohen et al. reported that hypokalemia is related to an increased risk of sudden cardiac death in hypertensive patients only when taking diuretics^13^. Diuretics not only aggravate hypokalemia but also promote neurohormonal activation^14^. This neurohormonal activation can also promote the severity of hypokalemia and may be related to a synergic effect between hypokalemia and diuretics to increase the risk of sudden cardiac death. In the present study, unfortunately, the number of patients with hypokalemia was too small to allow further subgroup stratifications according to diuretic use; thus, we could not evaluate the synergic effect between hypokalemia and diuretics on VSA prognosis. However, only one patient with prior diuretic use in the hypokalemia group experienced adverse events during follow-up. Moreover, while multivariate analysis showed that hypokalemia independent of prior diuretic use predicted adverse clinical outcomes in patients with VSA, prior diuretic use was not associated with our study outcomes. Therefore, we can carefully assume that hypokalemia-associated poor prognosis in patients with VSA was independent of the prior use of diuretics. The present study analysed only the impact of admission potassium levels because we had no data on serial measurement or discharge potassium levels. However, potassium levels might not significantly change during hospitalisation without active supplementation, acute deterioration of medical conditions, or use of drugs that affect potassium levels. A previous study of acute myocardial infarction also reported that potassium values tended to remain fairly constant during hospitalisation^4^. Although the present study showed that hypokalemia at admission was related to adverse outcomes in patients with VSA, future studies should investigate prolonged hypokalemia during hospitalisation or hypokalemia with or without diuretics and the associated adverse outcomes.

The most important question regarding the use of hypokalemia as a poor prognostic factor in patients with acute medical illness is whether hypokalemia is causally related to poor prognosis or is a surrogate marker of the severity of underlying diseases. Especially in patients with cardiovascular disease, the results of previous studies have suggested that potassium homeostasis may have a causal relationship with poor prognosis. The maintenance of serum potassium level decreases blood pressure through several mechanisms and hypokalemia is related to the incidence and outcome of hypertension^2,15,16^. These observations are also supported by several epidemiologic studies^17,18^. Furthermore, hypokalemia is related to life-threatening arrhythmia or death in patients with acute myocardial infarction or heart failure^4,19^. However, there have been no reports of a relationship between hypokalemia and VSA prognosis. In this study, we demonstrated that hypokalemia at admission was related to adverse cardiovascular outcomes in patients with VSA. Although the mechanism for these results is unclear, there are several possible explanations. Serum potassium has a vasodilator function by stimulation of membrane Na^+^, K^+^-adenosine triphosphatase activity, resulting in hyper-polarisation and relaxation of the vascular smooth muscle cell^20^. There was also report about potassium is involved in nitric oxide production^21^. In addition, increasing potassium levels had a beneficial effect on vascular endothelial cells through inhibition of oxygen-free radial formation^22^. Impaired vascular smooth muscle cell function, nitric oxide production, and vascular endothelial function were crucial factors associated with VSA aggravation^7^. Accordingly, hypokalemia may mediate vascular or endothelial dysfunction as the main mechanism of the relationship between hypokalemia and adverse cardiovascular outcome in patients with VSA.

Magnesium deficiency is a well-known electrolyte imbalance involved in VSA pathogenesis^23^. It is also related to VSA severity^24,25^. In general, habitual alcohol consumption in patients with VSA is assumed to promote the urinary excretion of magnesium, leading to tissue magnesium deficiency and coronary spasm^26^. Hypokalemia is a common electrolyte imbalance in patients with magnesium deficiency, occurring in 40–60% of patients^27^. In the present study, although we had no data on magnesium levels, there is possibility that hypokalemia may have been accompanied by hypomagnesemia, which may have affected our results. A previous epidemiologic study showed that the magnesium supplementation was inversely correlated with mortality of ischemic heart disease, however, it did not establish a relationship between magnesium levels and VSA prognosis^28^. Magnesium deficiency is causally related to alcohol consumption. In this study, alcohol consumption was not associated with either the incidence of hypokalemia or prognosis of VSA. It might be indirect evidence that hypokalemia may be a poor prognostic factor for VSA independent of hypomagnesemia. In present study, hyponatremia was the strongest independent prognostic factor for the VSA outcome. Moreover, hypertension was an independent predictor of hyponatremia and thiazide diuretic use had insignificant trend, but hypokalemia was not a predictor of hyponatremia (see Supplementary Table S1 online). Only three patients had both hyponatremia and hypokalemia, and primary outcomes did not occur in these patients. It can be possible evidence that both hyponatremia and hypokalemia were independent predictors of primary outcomes. However, we do not have a clear explanation of the relationship between hyponatremia and the outcome of VSA. Although there have been several reports about hyponatremia as an independent predictor of clinical outcomes in hospitalized patients, we could not find any report to provide evidence of an association between hyponatremia and the outcome of VSA^29,30^. There is a possibility that hyponatremia might be a strong surrogate marker of the severity of underlying medical illness, and it has a causal reason of relationship between hyponatremia and clinical outcomes. On the other hand, we should be carefully considered that the relationship between hyponatremia and diuretic use or hypertension has some impact on the prognosis of VSA. However, there were only 29 patients with hyponatremia (2.0%) in the present study which obviously limited the possibilities of in-depth analysis to clarify these hypotheses. Therefore, further studies with a large sample size focused on the association between hyponatremia and the prognosis of patients with VAS is needed.

Previous studies showed U-shape relationships between serum potassium level and mortality in acute medical illness^1,4^. In addition, there was concern regarding the benefit of maintaining high normal potassium levels in patients with heart failure or myocardial infarction^31^. We categorised patients into five different potassium groups: ≤ 3.5; 3.6– ≤ 4; 4.1– ≤ 4.5; 4.6– ≤ 5; and > 5 mEq/L and evaluated whether there was a U-shaped relationship between potassium levels and primary outcomes. Although the number of patients was small, we observed no event in patients with potassium levels > 5 mEq/L. Primary outcomes were most common in patients with potassium levels ≤ 3.5 mEq/L and least common in patients with potassium levels of 4.1–4.5 mEq/L. We observed no U-shaped relationship between potassium level and the incidence of primary outcomes or hazard ratio for the primary outcomes in the present study.

This study has several limitations. First, owing to its observational nature, confounding factors or unmeasured variables may have influenced the results. In addition, there was a possibility that patients in the hypokalemia group may have been more likely to have medical deterioration or chronic medical illnesses. Second, the hypokalemia group included only 70 patients and there were differences baseline characteristics between the hypokalemia and no-hypokalemia groups. We tried to mitigate the bias by applying multivariate tests; however, the small sample sizes may have caused bias. Third, this study did not capture serial potassium levels during hospitalisation; thus, we cannot evaluate the relationship between serial or discharge potassium levels and primary outcomes. Furthermore, we had no data on magnesium levels or discharge information regarding diuretics, which are important co-variables in potassium physiology. Fourth, the factors associated with frequency or severity of VSA symptoms, such as episodes of angina, incidence of visiting the emergency department due to chest pain, frequency of use of nitroglycerin, or escalation of anti-angina medications, which are important indicators for the evaluation of VSA outcomes, were not assessed. A previous study that assessed using magnesium levels showed that hypomagnesemia was related to VSA severity; however, the present study only demonstrated a relationship between hypokalemia and clinical outcome of VSA^24,25^. Finally, we could not further evaluate the potential significant impacts from hyponatremia because of small sample size. Therefore, the results of the present study should be interpreted cautiously.

## Conclusions

To our knowledge, this is the first study to show the association between hypokalemia and adverse cardiovascular outcomes of VSA in a large prospective cohort. The overall incidence of hypokalemia was 5% in patients with VSA and hypertension and prior thiazide diuretic use were risk factors for hypokalemia. Hypokalemia at admission was an independent predictor of adverse cardiovascular outcomes in VSA patients and hypokalemia may mediate vascular or endothelial dysfunction related to adverse outcomes. Therefore, further studies are warranted to determine whether maintaining adequate potassium levels by correcting for causal factors of hypokalemia or dietary supplementation affect the clinical outcomes of VSA.

## Methods

### Participants and cohort recruitment

The Variant Angina Korea (VA-Korea) registry is a nation-wide prospective, observational, and multicentre registry designed to reflect real-world clinical data of Korean patients with VSA. The study design and primary results of the registry have been published elsewhere^32–34^. Briefly, a total of 2960 patients with chest pain and suspected VSA who underwent coronary angiography and ergonovine (EG) provocation test at 11 tertiary hospitals in Korea were consecutively enrolled from May 2010 to June 2015. Patients with known malignant or systemic inflammatory disease, end-stage renal disease on dialysis, and catheter-induce spasms at baseline coronary angiography did not include the registry. The study protocol was approved by the institutional review board of each participating hospital including Hallym University Sacred Heart Hospital Institutional Review Board, named ‘HUMC ethics committees & IRB’ (IRB No. 2010-I007), and all patients provided written informed consent. The study complied with the principles of the Declaration of Helsinki.

### Coronary angiography and provocation test for VSA

An EG provocation test was performed and the diagnosis of VSA was confirmed based on VSA diagnosis and treatment guidelines^7^. The vasodilatory drugs including nitrates, calcium channel blockers, and nicorandil were withheld at least 48 h before coronary angiography. After performing baseline coronary angiography, a spasm provocation test was performed with intracoronary EG injection on the left coronary artery. The EG was mixed with saline and administered by intracoronary bolus injection. Incremental doses of 20 μg (E1), 40 μg (E2), and 60 μg (E3) of EG were injected into the left coronary artery. In cases with a negative result in the left coronary artery, incremental doses of 10 μg (E1), 20 μg (E2), and 40 μg (E3) of EG were injected into the right coronary artery. The definition of a positive result on the EG provocation test was ≥ 50% luminal diameter narrowing during the test with or without ischemic symptoms or electrocardiographic (ECG) changes. Among the positive results, total occlusion or > 90% luminal diameter narrowing of the coronary artery accompanied by ischemic symptoms or ECG changes was classified as definite VSA, while 50–90% luminal narrowing with or without ischemic symptoms or ECG changes was classified as possible VSA. Negative results were defined as < 50% luminal narrowing without ischemic symptoms and ECG changes. A focal spasm was defined as a discrete luminal narrowing in one coronary segment, whereas a diffuse spasm was defined as the luminal narrowing observed continuously from the proximal to the distal segments of the coronary arteries. Underlying atherosclerotic stenosis was measured after full dilatation of the coronary arteries via the intracoronary injection of nitroglycerine, in which ≥ 50% atherosclerotic stenosis of one of the major epicardial arteries was defined as indicative of obstructive coronary artery disease.

### Definitions and endpoints

Hypokalemia was defined as serum potassium ≤ 3.5 mEq/L and hyponatremia as ≤ 135 mEq/L on admission laboratory testing. The patients were divided into hypokalemia and no-hypokalemia groups according to serum potassium level. We also categorised patients into five different potassium groups: ≤ 3.5; 3.6– ≤ 4; 4.1– ≤ 4.5; 4.6– ≤ 5; and > 5 mEq/L. Hypertension was defined as blood pressure ≥ 140/90 mmHg or currently using anti-hypertensive medications. Diabetes mellitus was defined as glycated haemoglobin level ≥ 6.5% or currently using oral hypoglycaemic agents/insulin. Chronic kidney disease was defined as a glomerular filtration rate less than 60 mL/min per 1.73 m^2^. Habitual drinking was defined as drinking more than three times per week regardless of the quantity of alcohol consumed. The primary outcome was a composite of cardiac death, acute coronary syndrome (ACS) including myocardial infarction, and new-onset life-threatening arrhythmia such as ventricular tachycardia/fibrillation or complete atrioventricular block during the follow-up period. Cardiac death was defined as death with a demonstrated cardiac cause such as myocardial infarction, fatal arrhythmia, or heart failure or sudden unexplained death without evidence of a non-cardiac cause. ACS was defined as prolonged ischemic chest pain lasting for more than 20 min with evidence of ischemic ECG changes and/or elevated cardiac troponin I. All adverse events of interest were adjudicated by the Local Events Committee of Seoul St. Mary’s Hospital through source document review.

### Statistical analysis

Continuous variables were expressed as means ± standard deviation and mean differences between groups were estimated using *t-*tests. Categorical variables were expressed as numbers and their percentages and differences between groups were estimated using *χ*^2^ tests. Logistic regression analysis was used to determine the predictors of hypokalemia. The cumulative event rate was assessed using the Kaplan–Meier method with log-rank analysis. Multivariable Cox regression analysis was used to evaluate the adjusted relative risks of the variables. The multivariable models included age; sex; hypertension; diabetes; chronic kidney disease; renin-angiotensin system inhibitors and thiazide diuretic use at admission; spasm characteristics; the prescription of aspirin, calcium channel blocker, nitrates, and statin at discharge; hyponatremia; and hypokalemia. These variables were chosen according to their clinical relevance and were based on the results of previous trials^7,33^. All statistical analyses were performed using IBM SPSS Statistics for Windows, version 26.0 (IBM Corp., Armonk, NY, USA), with *P* values < 0.05 considered statistically significant.

## Supplementary Information

Supplementary Information.

## Data Availability

The datasets generated during and/or analysed during the current study are available from the corresponding author on reasonable request.
