# Supplementary material for: Admission serum potassium levels and prognosis of vasospastic angina
Source: Sci Rep. 2021 Mar 11;11:5707. doi: 10.1038/s41598-021-84712-w (PMC7952915; doi:10.1038/s41598-021-84712-w)
Supplement: Supplementary file 1 — Supplementary Information. [file 41598_2021_84712_MOESM1_ESM.pdf]

Supplement for "**Admission Serum Potassium Levels and Prognosis of Vasospastic Angina**"

Authors: Won-Woo Seo, Sang-Ho Jo, Sung Eun Kim, Hyun-Jin Kim, Seung Hwan Han, Kwan Yong Lee, Sung Ho Her, Min-Ho Lee, Sung Seek Cho, Hack-Lyoung Kim, Sang Hong Baek

Supplementary Table S1. Predictor of hyponatremia in patients with vasospastic angina

Variable	Odds ratio	95% CI	P value
Age ≥60 years	1.18	0.52 − 2.69	0.694
Male	1.28	0.51 – 3.23	0.606
Hypertension	2.59	1.06 − 6.34	0.037
Diabetes	0.77	0.22 − 2.68	0.677
Chronic kidney disease	2.08	0.77 – 5.63	0.150
Habitual drinking	1.80	0.73 – 4.43	0.204
RAS inhibitor at admission	0.96	0.37 − 2.47	0.931
Thiazide diuretics at admission	2.64	0.87 − 8.04	0.087
Beta-blocker at admission	0.93	0.26 – 3.34	0.905
Hypokalemia	1.71	0.48 – 6.10	0.407
Definite spasm	1.12	0.51 − 2.44	0.784
Focal spasm	0.73	0.32 − 1.64	0.441

RAS, renin-angiotensin system
